# Outcome of Patients With Esophageal Atresia and Very Low Birth Weight (≤ 1,500 g)

**DOI:** 10.3389/fped.2020.587285

**Published:** 2020-11-17

**Authors:** Laura Antonia Ritz, Anke Widenmann-Grolig, Stefan Jechalke, Sandra Bergmann, Dietrich von Schweinitz, Eberhard Lurz, Jochen Hubertus

**Affiliations:** ^1^Department of Pediatric Surgery, Dr. von Hauner Children's Hospital, Ludwig Maximilian University of Munich, Munich, Germany; ^2^KEKS e.V., National German Patient Organization for Diseases of the Esophagus, Stuttgart, Germany; ^3^Department of Pediatric Surgery, Speech and Language Therapy, Dr. von Hauner Children's Hospital, Ludwig Maximilian University of Munich, Munich, Germany; ^4^Department of Pediatric Gastroenterology, Dr. von Hauner Children's Hospital, Ludwig Maximilian University of Munich, Munich, Germany

**Keywords:** esophageal atresia, very low birth weight (VLBW), extremely low birth weight (ELBW), long-term outcome, pediatric surgery, primary repair, staged repair

## Abstract

**Introduction:** Primary repair of esophageal atresia (EA) in infants with very low birth weight (VLBW) and extremely low birth weight (ELBW) has been widely performed in pediatric surgery. However, several studies have shown that complication rates in infants with VLBW are high. We hypothesize preterm children benefit from a shorter, less-traumatizing operation in the first days of life, as staged repair implies.

**Methods:** Patients with EA and VLBW were retrieved from the database of a large national patient organization KEKS e.V. Structured questionnaires were sent to all the patients' families; the responses were pseudonymized and sent to our institution.

**Results:** Forty-eight questionnaires from patients were analyzed. The mean birth weight was 1,223 g (720–1,500 g). Primary repair was performed in 25 patients (52%). Anastomotic insufficiency (AI) was reported in 9 patients (19%), recurrent fistula (RF) in 8 (17%), and anastomotic stenosis in 24 patients (50%). Although AI was almost twice as common after primary repair than after staged repair (24 vs. 13%; *p* = 0.5), the difference was not statistically significant. RF was more frequent after primary repair (28 vs. 4%; *p* = 0.04), gastroesophageal reflux was more frequent in the group after staged repair (78 vs. 52%; *p* = 0.04), and both correlations were statistically significant. Intracranial hemorrhage (ICH) was reported in 11 patients (23%) and was observed in 7 of them (64%, *p* = 0.4) after primary repair. ICH was reported in 60% of patients with ELBW and 75% of patients when ELBW was paired with primary repair.

**Conclusion:** This study demonstrates the complication rate in patients with VLBW is higher than the average of that in patients with EA. The study indicates that a staged approach may be an option in this specific patient group, as less RF and AI are seen after staged repair. ICH rate in patients with ELBW seemed to be especially lower after staged repair. Interestingly, gastroesophageal reflux was statistically significantly higher in the group after staged repair, and postoperative ventilation time was longer. It is therefore necessary to individually consider which surgical approach is appropriate for this special patient group.

## Introduction

Esophageal atresia (EA) is the most common congenital anomaly of the esophagus and causes interrupted esophageal continuity and usually results in a tracheoesophageal fistula (TEF) (1). EA repair is a complex operation, even in full-term neonates (2), and has a negative impact on blood circulation and respiration, causing extra perioperative stress (3, 4). In the case of patients with very low birth weight (VLBW), the operation is aggravated by several other factors. Preterm babies with VLBW present with cardio-pulmonary vulnerability, an immature immune system, and the risk of preterm complications such as necrotizing enterocolitis and intracranial hemorrhage (ICH), to mention but a few (5–7).

On the other hand, delayed repair can be physically demanding, especially because TEF impedes respiration and bears the risk of recurrent aspiration of gastric juice. Finally, enteral feeding is not possible in these patients; therefore, prompt ligation of the fistula should be pursued within the first 48 h of life (8) to enable enteral feeding. In this paper, we mainly discuss two different treatment strategies for patients with EA and VLBW. The first is primary repair—in which case, ligation of a possible TEF and esophageal anastomosis are performed in the same session. Even if only one operation is needed to establish the continuity of the esophagus, operation time, and anesthesia time are longer. In addition, esophageal tissue is very vulnerable and thin in VLBW, complicating the esophageal anastomosis (5). The second is staged repair, which might be a reasonable alternative for infants with VLBW. In this case, gastrostomy is performed during the first operation to enterally feed the child, and TEF, if present, is closed (9). Esophageal anastomosis is delayed until the patient stabilizes and gains sufficient weight, usually >2,000 g.

This study aimed to analyze the treatment outcome of patients born with VLBW—focusing on operative strategies and the appearance of operation-related complications while hypothesizing preterm children benefit from a shorter and less-traumatizing operation in the first days of life, as staged repair implies.

## Materials and Methods

Patients born with all types of EA and VLBW (≤1,500 g) were included in this study. A list of a subgroup of patients with extremely low birth weight (ELBW; ≤1,000 g) was also generated. Exclusion criteria were birth weight >1,500 g and insufficient dataset.

The data of the infants were retrieved from the German patient support group for patients with diseases of the esophagus (KEKS e.V.). A structured questionnaire was created to gather information about the surgical approach, hospital stay, and clinical outcome, as was done in other studies (10). Questions asked were familiar to the patients/parents because they were based on the questions asked in the KEKS e.V. follow-up folder. KEKS e.V. sent the questionnaires in April 2019 and the responses were pseudonymized and sent to our institution (Dr. von Hauner Children's Hospital, Department of Pediatric Surgery, Ludwig Maximilian University of Munich, Germany) between June 2019 and January 2020. The questionnaire addressed two demographic items, eight questions on clinical features, eight questions on clinical/surgical management, and nine healing-process items (Supplement 1 details this questionnaire). If available, pseudonymized medical reports were also provided for further analysis.

Accompanied by the questionnaires, we obtained written informed consent from all the families included in this study. The ethics committee of the Ludwig Maximilian University of Munich, Germany, approved the study (reference number 18–585). In collaboration with the Institute for Medical Information Processing, Biometry, and Epidemiology of the Ludwig Maximilian University of Munich, Germany, we performed the statistical analysis using SPSS (IBM® SPSS Statistics, version 25).

**Figure 1:**
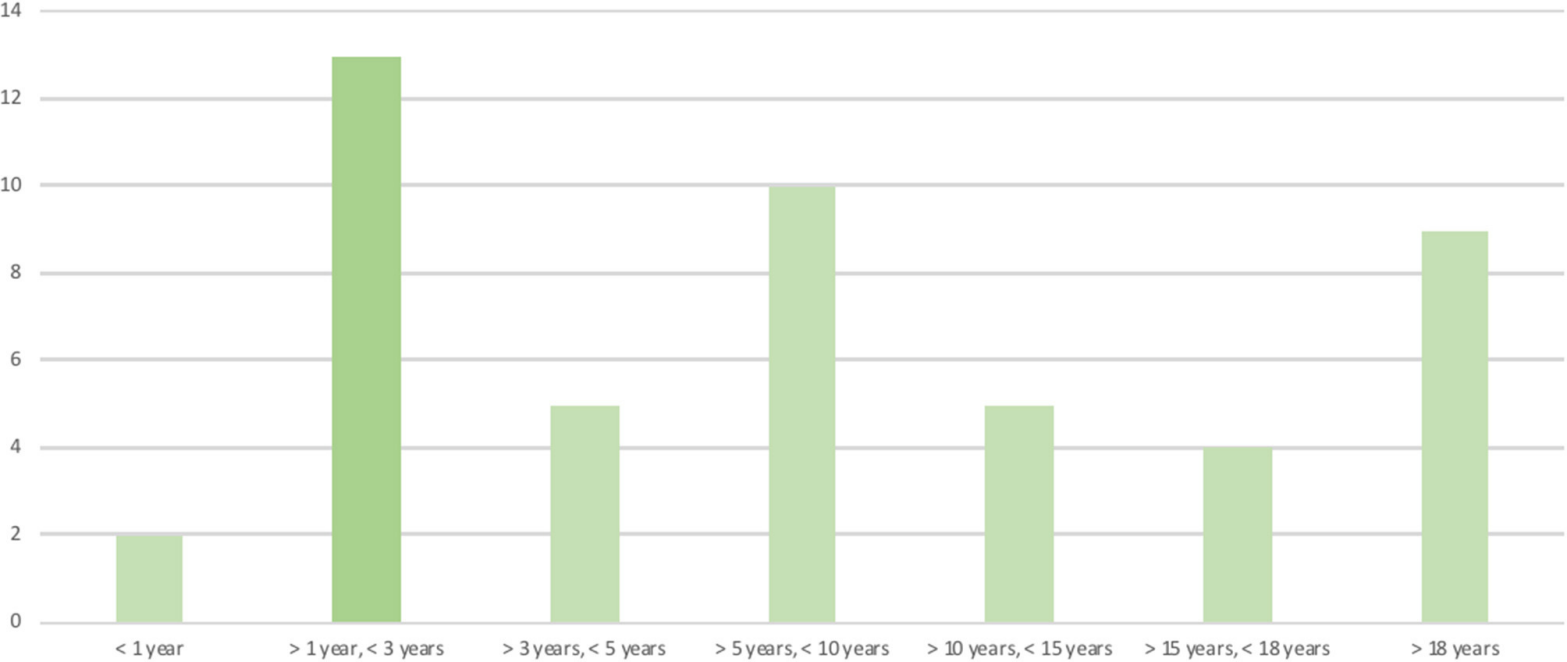
Patient's age at the time the questionnaire was answered.

The patients' ages at the time the questionnaires were answered were clustered in age groups (Figure 1).

Questions on the clinical outcome followed the questions asked and explained in the KEKS e.V. follow-up folder. AI was defined as leaking at the area of the anastomosis; anastomotic stenosis (AS) as the reduced diameter of the area of the anastomosis, requiring dilatation or causing food-bolus sticking; and gastroesophageal reflux (GER) as having symptoms of heartburn, stains of saliva with hematin on the pillow, coughing, and acid-smelling breath.

The patients were divided into groups treated with primary, delayed primary, and staged repairs. Delayed primary repair describes ligation of the fistula and anastomosis of the esophagus, with the same operation taking place after more than 7 days of life. We started with multiple linear regression with backward elimination—followed by Kendall Tau-b test, chi-square test, ANOVA test, and Mann–Whitney *U*-two-sample rank-sum test to identify the indicators for the chosen surgical approach, as well as the possible risk factors for the occurrence of complications. Finally, we performed a subgroup analysis where we described the influence of birth weight and the EA type on the treatment outcome. *P* < 0.05 was defined as statistically significant.

## Results

**Figure 2:**
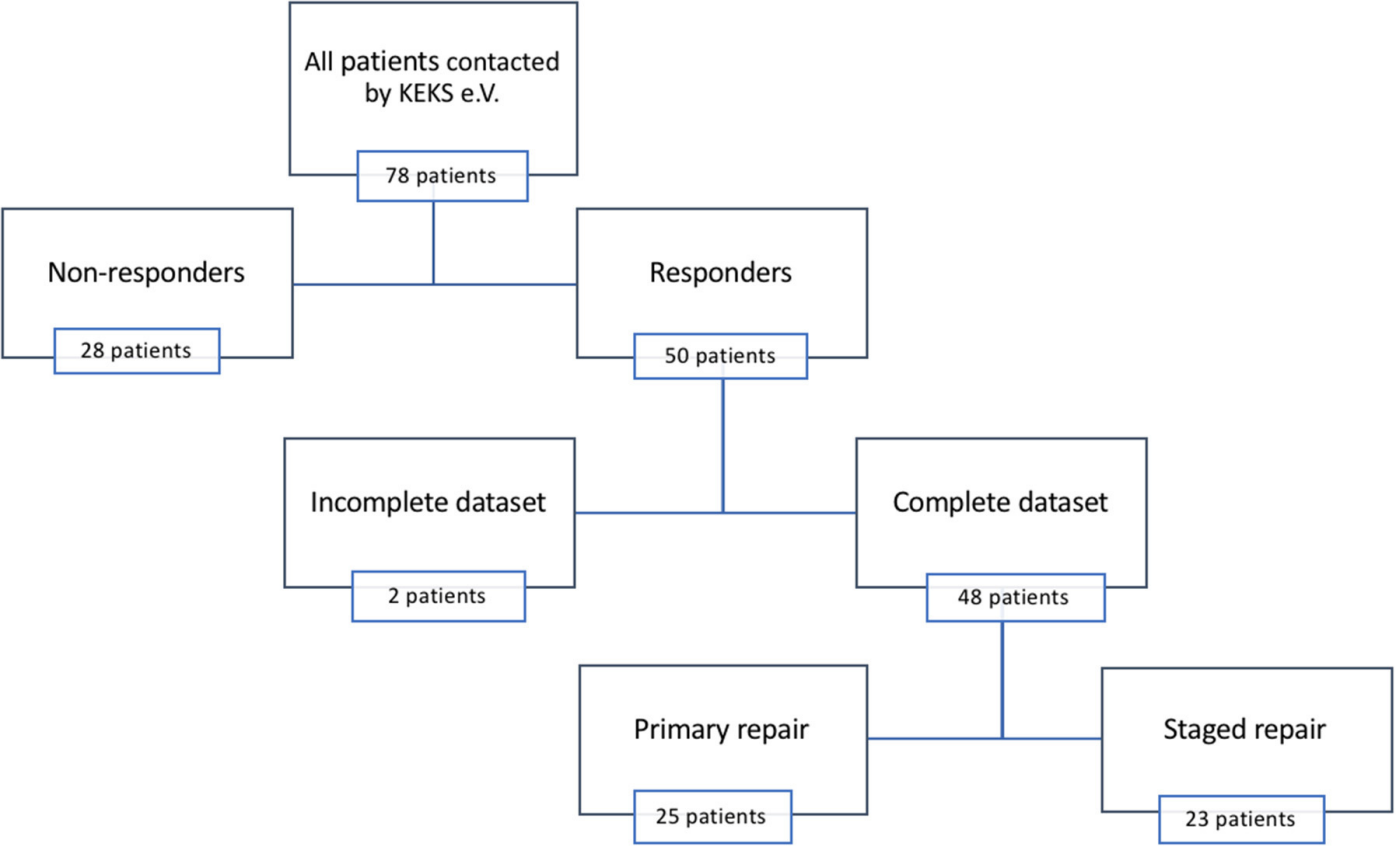
Drop-out analysis.

Questionnaires were sent to 78 patients and their parents, and we recorded a two-third return rate (*n* = 50). Two incomplete datasets were excluded from further analysis (Figure 2). Gestational age ranged from 28 to 35 weeks (mean = 31 weeks, interquartile range [IQR] = 3), while birth weight ranged from 720 g to 1,500 g (mean = 1,223 g; IQR = 391). Twenty patients (42%) provided their medical reports. Full epidemiological data are listed in Tables 1, 2.

**Table 1:** Epidemiological data.

		**All**	**Primary**	**Staged**	***P*-value^a^**
Male	Yes	21	8 (38%)	13 (62%)	0.09
	No	27	17 (63%)	10 (37%)	
ELWB	Yes	10	4 (40%)	6 (60%)	0.4
	No	38	21 (55%)	17 (45%)	
EA type/Gross	A	7	0 (0%)	7 (100%)	***0.01***
	B	1	1 (100%)	0 (0%)	
	C	37	21 (67%)	16 (43%)	
	D	2	2 (100%)	0 (0%)	
	Unknown	1	1 (100%)	0 (0%)	
Congenital heart disease	Yes	16	9 (56%)	7 (44%)	0.7
	No	30	15 (50%)	15 (50%)	
	Unknown	2	1 (50%)	1 (50%)	
Heart function	Normal	41	21 (51%)	20 (49%)	0.9
	Affected	5	3 (60%)	2 (40%)	
	Unknown	2	1 (50%)	1 (50%)	
VACTERL association	Yes	11	6 (55%)	5 (45%)	0.9
	No	35	18 (51%)	17 (49%)	
	Unknown	2	1 (50%)	1 (50%)	
Congenital anomalies	Yes	21	12 (57%)	9 (43%)	0.7
	No	25	11 (44%)	14 (56%)	
	Unknown	2	2 (100%)	0 (0%)	
Intracranial haemorrhage	Yes	11	7 (64%)	4 (36%)	0.4
	No	35	17 (49%)	18 (51%)	
	Unknown	2	2 (100%)	0 (0%)	
Preoperative ventilation	Yes	33	17 (52%)	16 (48%)	0.5
	No	11	7 (64%)	4 (36%)	
	Unknown	4	1 (25%)	3 (75%)	
Time to fistula closure	<24h	11	7 (64%)	4 (36%)	0.8
	>24 h, <48 h	9	7 (78%)	2 (22%)	
	>48 h, <5 d	8	4 (50%)	4 (50%)	
	>5 d, <7 d	2	1 (50%)	1 (50%)	
	>7d No fistula	8 7	5 (63%) 0 (0%)	3 (37%) 7 (100%)	
	Unknown	3	1 (33%)	2 (67%)	
Postoperative ventilation	Yes	40	23 (58%)	17 (42%)	
	Unknown	8	2 (25%)	6 (75%)	
No. of operations	1	9	9 (100%)	/	***0.02***
	2	11	5 (45%)	6 (55%)	
	3	4	2 (50%)	2 (50%)	
	> 3	21	8 (38%)	13 (62%)	
	Unknown	3	1 (33%)	2 (67%)	
AI	Yes	9	6 (67%)	3 (33%)	0.5
	No	29	15 (52%)	14 (48%)	
	Unknown	10	4 (40%)	6 (60%)	
RF	Yes	8	7 (88%)	1 (12%)	***0.02***
	No	27	12 (44%)	15 (56%)	
	Unknown	13	6 (46%)	7 (54%)	
AS	Yes	24	14 (58%)	10 (42%)	0.5
	No	13	6 (46%)	7 (54%)	
	Unknown	11	5 (45%)	6 (55%)	
Gastrostomy	Yes	28	8 (29%)	20 (71%)	***0.01***
	No	16	14 (88%)	2 (12%)	
	Unknown	4	3 (75%)	1 (25%)	
GER	Yes	31	13 (42%)	18 (58%)	***0.02***
	No	12	10 (83%)	2 (17%)	
	Unknown	5	3 (60%)	2 (40%)	

*This table shows all the information collected. Statistical correlation of primary repair and each category (e.g., EA type: A,B, C, D) was tested*.

* *The p-value relates to the category, not the single parameter in a category, and is calculated without unknown cases performing chi-square test. Statistically significant p-values are underlined and marked in bold*.

*EELBW, Extremely low birth weight; EA, Esophageal atresia; VACTERL, Vertebral, intestinal atresia, cardiac, tracheal, renal, limb malformations; No., Number; AI, Anastomotic insufficiency; RF, Recurrent fistula; AS, Anastomotic stenosis; GER, Gastroesophageal reflux*.

**Table 2:** Outcome after primary vs. staged repair in the different subgroups.

**Subgroup**			**AI**	**RF**	**AS**	**GER**	**1 surg**.	**2 surg**.	**3 surg**.	**> 3 surg**.	**ICH**
All patients	All	48 (100%)	9 (19%)	8 (17%)	24 (50%)	31 (65%)	9 (18%)	11 (23%)	4 (8%)	21 (44%)	11 (23%)
	Primary	25 (100%)	**6 (24%)**	**7 (28%)**	14 (56%)	13 (52%)	**9 (36%)**	5 (20%)	2 (8%)	8 (32%)	**7 (28%)**
	Staged	23 (100%)	3 (13%)	1 (4%)	10 (43%)	**18 (78%)**	/	6 (26%)	2 (9%)	**13 (57%)**	4 (17%)
VLBW + ELBW + Type C	All	37 (100%)	6 (16%)	6 (16%)	18 (48%)	21 (57%)	9 (24%)	9 (24%)	3 (8%)	15 (40%)	**11 (30%)**
	Primary	21 (100%)	**5 (24%)**	**5 (24%)**	**12 (57%)**	5 (48%)	**9 (43%)**	5 (24%)	1 (5%)	5 (24%)	7 (33%)
	Staged	16 (100%)	1 (6%)	1 (6%)	6 (37%)	**11 (69%)**	/	4 (25%)	2 (12%)	**10 (62%)**	4 (25%)
eVLBW	All	38 (100%)	7 (18%)	7 (18%)	17 (45%)	24 (63%)	8 (21%)	8 (21%)	4 (10%)	15 (40%)	5 (13%)
	Primary	21 (100%)	**6 (29%)**	**6 (29%)**	10 (48%)	10 (48%)	**8 (38%)**	4 (19%)	2 (10%)	6 (29%)	**4 (19%)**
	Staged	17 (100%)	1 (6%)	1 (6%)	7 (41%)	**14 (82%)**	/	4 (24%)	2 (12%)	**9 (53%)**	1 (6%)
eVLBW + Type C	All	28 (100%)	5 (18%)	5 (18%)	12 (43%)	15 (54%)	8 (28%)	6 (21%)	3 (11%)	10 (35%)	5 (18%)
	Primary	18 (100%)	**5 (28%)**	**4 (22%)**	9 (50%)	8 (44%)	8 (44%)	4 (22%)	1 (6%)	4 (22%)	**4 (22%)**
	Staged	10 (100%)	0 (0%)	1 (10%)	3 (30%)	**7 (70%)**	/	2 (20%)	2 (20%)	**6 (60%)**	1 (10%)
ELBW	All	10 (100%)	2 (20%)	1 (10%)	7 (70%)	7 (70%)	1 (10%)	3 (30%)	0 (0%)	6 (60%)	6 (60%)
	Primary	4 (100%)	0 (0%)	1 (25%)	4 (100%)	3 (75%)	1 (25%)	1 (25%)	0 (0%)	2 (50%)	3 (75%)
	Staged	6 (100%)	2 (33%)	0 (0%)	3 (50%)	4 (67%)	/	2 (33%)	0 (0%)	4 (67%)	3 (50%)
Type A	All	7 (100%)	1 (14%)	/	**4 (57%)**	**7 (100%)**	/	2 (29%)	0 (0%)	3 (43%)	**0 (0%)**

*AI, Anastomotic insufficiency; RF, Recurrent fistula; AS, Anastomotic stenosis; GER, Gastroesophageal reflux; Surg., Surgery; ICH, Intracranial hemorrhage; VLBW, Very low birth weight (≤ 1,500 g); ELBW: Extremely low birth weight (≤ 1,000 g); eVLBW, Exclusively VLBW (>1,000 g; ≤1,500 g)*.

EA Gross type C was predominant—with 37 cases (77%), followed by type A with 7 cases (15%), type D with 2 cases (4%), and type B with 1 case (2%). Primary and delayed primary repair was performed in 25 patients (52%). Staged repair was performed in 23 patients (48%). Twenty-five patients (52%) needed three or more surgeries in total. ICH was seen in 11 patients (23%): I° in 5 (10%) and II° and III° in 3 patients (6%) each.

AI was reported in 9 patients (19%), recurrent fistula (RF) in 8 (17%), and AS in 24 patients (50%), while 19 patients (40%) needed more than three dilations in the first 2 years of life. GER was reported in 31 patients (64%), and 27 patients (55%) were using proton-pump inhibitors. Fundoplication was performed in five patients (10%) within the first year of life.

### Primary Repair vs. Staged Repair

There was no statistically significant difference in the gender distribution between primary repair and staged repair (*p* = 0.09). Similarly, there was no statistically significant difference in preoperative (*p* = 0.5) or postoperative mechanical ventilation (*p* = 0.9). However, there was an enormous range of ventilation days in both groups (primary repair: median = 10 days [range = 4–270 d; IQR = 21], staged repair: median = 13 days [range = 2–120 d; IQR = 45]). ICH was seen in seven patients (28%) after primary repair and in four patients (17%) after staged repair (*p* = 0.4).

Although AI was almost twice as common after primary repair than after staged repair (24 vs. 13%, *p* = 0.5), the difference was not statistically significant. AS showed no statistically significant difference in both groups (56 vs. 43%). However, RF was significantly more frequent after primary repair statistically (28 vs. 4%; *p* = 0.04). GER was more frequent in the group after staged repair (78 vs. 52%), and the difference was statistically significant (*p* = 0.04; Tables 1, 2).

Linear regression with backward elimination was performed for different dependent and independent variables. The analysis revealed a correlation between surgical approach and EA type, birth weight, and duration of postoperative mechanical ventilation, AS and EA type, as well as AS and AI. Therefore, a subgroup analysis of EA types and birth weight was performed, detailed in the following paragraphs, to show the impact of these factors on the treatment outcome (Table 2).

### Birth Weight

To investigate the influence of birth weight on the treatment outcome, exclusively VLBW (eVLBW: birth weight >1,000 and ≤1,500 g) and ELBW (birth weight ≤1,000 g) were analyzed separately (Table 2). Even though 100% of patients with ELBW and 64% of patients with eVLBW were ventilated preoperatively, this was not statistically significant (*p* = 0.2). However, there was a statistically significant difference in the duration of postoperative ventilation. While patients with eVLBW were ventilated for 9 days, this value increased to 30 days in patients with ELBW (*p* = 0.04). Patients with ELBW were ventilated particularly long after staged repair (median ventilation time = 60 days). While complications such as AI and RF did not seem to be affected by birth weight, AS and ICH occurred more often in patients with ELBW, especially after primary repair. AS increased from 48% in patients with eVLBW to 100% in patients with ELBW (*p* = 0.2) after primary repair, while the occurrence of ICH went from 19% in patients with eVLBW to 75% in patients with ELBW (*p* = 0.07) after primary repair.

## Discussion

Haight et al. (11) published their experience on primary repair of EA in 1944, and this approach soon became the surgical standard (3). Nevertheless, mortality remained high, especially in preterm neonates. To identify patients with EA with a high risk of mortality, different risk classifications were introduced. In these classifications, birth weight, and concomitant health issues played a variable role (12–14). With improvements in surgical technique, pediatric anesthesiology, and neonatal care, primary repair became technically possible, even in preterm babies (1, 3, 4). Almost 20 years ago, Chahine and Ricketts (5) published a review paper and stated it was no longer necessary to perform staged repair in patients born with EA and VLBW. Since then, several studies advocating either primary or staged repair with different conclusions have been published [(15–25); Table 3].

**Table 3:** Literature on esophageal atresia (EA) repair in very low birth weight (VLBW)/extremely low birth weight (ELBW).

**Author**	**Year**	**Period**	**n^1^**	**Weight**	**Primary**	**Staged**	**EA Type**	**Aim of the study**	**Conclusion**
Schmidt et al. (15)	2017	2002–2016	11	4 ELBW 7 VLBW (24 > 1,500 g)	4 7 24		Gross C	Compare outcome after primary open repair in VLBW/ELBW with BW > 1,500 g	Complications are unrelated to bodyweight
Hannon et al. (16)	2016	1993–2015	9	ELBW (m^2^ = 815 g)	2	7	All	Outcome of EA repair in ELBW	56% survival, due to immaturity; Gross A with staged repair, 100% survival
Zani et al. (17)	2016	2000–2014	7	ELBW (m = 930 g)	1	6	All	Outcome of EA repair in ELBW	Complications associated with prematurity
Margain et al. (18)	2014	2012–2013	3	ELBW (m = 690 g)		3	Gross C	Outcome of EA repair in ELBW treated with LEB^3^ and delayed repair	LEB enables delayed repair (> 2100g)
Ito et al. (19)	2013		1	ELBW (471 g)		1	Gross C	Case report: Delayed primary repair after EB^4^	Complications associated with prematurity EA repair was performed too late
Petrosyan et al. (20)	2009	1987–2008	25	VLBW (m = 1380 g)	16	9	All	Comparison between primary and staged repair in EA	Significantly more complications after primary repair
Seitz et al. (21)	2006	2002–2004	4	VLBW (m = 920 g)	4		Gross C	Outcome of EA in VLBW	Primary repair is technically feasible, AI and AS in 25%
Chahine and Ricketts (5)	2000	1981–1999	10	VLBW (m = 1160 g)	1	9	all	Outcome of EA in VLBW	10% died after sepsis caused by AI GER and fundo in 62.5%
Driver et al. (22)	1997		1	ELBW (740 g)	1		Gross C	Case report	Good outcome due to improved NICU treatment
Alexander et al. (23)	1993	1966–1986	21	<2,000 g (1590 g)	4	17	all	Comparison between primary and staged repair in EA	Staged repair incurred a lesser morbidity
Schaarschmidt et al. (24)	1992		1	ELBW (445 g)		1		Delayed primary repair without ligation of TEF^5^, Case report	No operation-associated complications
Todd et al. (25)	1990		1	ELBW (700g)		1		Outcome of EA (IIIb) and delayed repair after LEB^3^, Case report	Mechanical ventilation after LEB^3^ dilatates the distal esophagus

*Overview of the literature regarding the repair of EA with or without TEF in patients born with exclusively ELBW and VLBW, without the claim of completeness*.

1 *n, number of patients*.

2 *m, median*.

3 *LEB, lower esophageal banding*.

4 *EB, esophageal banding*.

5 *TEF, tracheo-esophageal fistula*.

These studies are solitarily retrospective single-center analyses with a maximum of 25 patients. We hypothesized that, even if technically possible, primary repair might not always be the superior surgical approach for patients with EA and VLBW and that staged repair is a valid option and should be taken into consideration when dealing with these patients.

We analyzed 48 patients unified in the German patient support group for patients with diseases of the esophagus (KEKS e.V.), with a focus on operative strategy and appearance of surgery-related complications. Patients included in this study were treated in different German hospitals. EA Gross type C was predominant (77%, compared to 80–85% in the literature) (9, 11). In contrast, type A was almost twice as high as described in epidemiological data (15 vs. 8%) (9) but almost equal compared to other questionnaires sent to patient support groups (11), implying patients with EA and type A are more likely to participate in patient support groups.

### Surgical Approach

The distribution of primary and staged repairs was almost equal in our cohort. In patients with ELBW, the distribution changed to 40% for primary repair and 60% for staged repair. These findings imply ELBW might have an impact on the chosen surgical approach.

In general, our findings suggest patients with VLBW remain with a higher risk for the development of complications after EA repair compared to previously published data of patients with EA and all weight groups (10, 13, 26). In our cohort, AI was seen in 19%, compared to 7–8% in all patients with EA (10, 13). RF was reported in 17%, compared to 2–4%, and AS in 50%, compared to 22% in the literature on patients with EA (10, 13). This suggests the complication rate in patients with VLBW is generally higher than the average. But does the surgical approach also have an influence on the complication rate? Considering AI, the incidence rate in the group after primary repair was twice as high (24 vs. 13%) but not statistically significant. There was no statistically significant difference for AS, although it was very high in both groups overall (56 and 43%) compared to all patients with EA. However, there was a statistically significant difference for RF. While RF after staged repair occurred in only 4% of patients, this complication was seen in 28% after primary repair (*p* = 0.04). Therefore, staged repair seems to be superior to primary repair for patients with VLBW.

GER was reported in 52% of the patients. This goes in line with the literature (13, 26). After staged repair, GER was almost twice as high compared to after primary repair, with no vivid explanation.

These results are consistent with the data of Petrosyan et al. (20), who reported significantly more complications after primary repair, and Alexander et al. (23). Both studies stated that the immaturity of tissue and organs in patients with VLBW is responsible for the high numbers of surgery-associated problems (20, 23). In our data, there was no other parameter (e.g., heart function, VACTERL, and preoperative ventilation) leading to a higher risk for surgery-associated complications. Therefore, the immaturity of the tissue could be a possible explanation. This contrasts with the results of Seitz et al. (21), who shared their data on four patients with EA type C and VLBW treated with primary repair (AI and AS in 25% of their patients) in 2006. Nine years later, the researchers published their data with 11 patients and compared the outcome to open primary repair in patients with birth weight >1,500 g, stating anastomotic complications are not related to factors of tissue prematurity and birth weight (15). A possible explanation for these outstanding results may be because the university hospital has a standard structure for treating these complex cases, once more demonstrating the importance of centralization.

### Birth Weight

ELBW had an additional impact. While 64% of patients with eVLBW were ventilated preoperatively, the rate for ELBW was 100%, although not statistically significant. This difference is more pronounced in the postoperative phase. Here, patients with eVLBW were ventilated for “only” 9 days, whereas this value increased to 30 days in patients with ELBW. A particularly long ventilation time of 60 days was observed in patients with ELBW who underwent staged repair. A direct explanation for this cannot be read from the data. A dreaded complication of these vulnerable patients is ICH. Since the known risk factors for ICH—such as sepsis, increased interleukins, pneumothorax, and the use of catecholamines (27)—were not asked for in the questionnaire, these factors were not included in the analysis. However, it was found that 19% of patients with eVLBW developed ICH. This value increased to 60% in patients with ELBW. While 50% of patients with ELBW developed ICH after staged repair, the value in patients with ELBW after primary repair increased to 75%. Due to the very small number of patients in the individual groups, a statistical statement does not make sense here and is, therefore, only descriptive.

### Limitations

Since our data were from members of a patient support group, we could not describe the mortality rate. Furthermore, a selection bias exists, as only patients who were members of the KEKS e.V. patient support group participated and reported their personal experience in this study and we are unable to say how the included patients (64%) represent the population. Medical data were provided for <50% of the patients; for the remaining cohort, we had to rely on the questionnaire alone. The reliability of the answers given by the patients or their parents increases since the questions asked are familiar to them because they were based on the questions asked in the KEKS e.V. follow-up folder.

### Conclusion

This study shows the complication rate in patients with VLBW is higher than the average of patients with EA. The study indicates that a staged approach may be an option in this specific patient group. Thus, less RF and AI are seen after staged repair. Especially in patients with ELBW, there is evidence that the ICH rate after staged repair is lower. Interestingly, GER was significantly higher in the group after staged repair statistically, and postoperative ventilation time was significantly longer statistically. For this very special patient group, it is therefore necessary to individually consider which surgical approach is appropriate. To improve data on this issue, prospective studies with a higher number of patients are needed.

## Data Availability Statement

The raw data supporting the conclusions of this article will be made available by the authors, without undue reservation.

## Ethics Statement

The studies involving human participants were reviewed and approved by Ethics committee of the Ludwig-Maximilian's University Munich, Germany (Reference number 18-585). Written informed consent to participate in this study was provided by the participants' legal guardian/next of kin.

## Author Contributions

LR, JH, and EL contributed in an equal manner as the main authors of this article. All authors contributed to the article and approved the submitted version.

## Conflict of Interest

The authors declare that the research was conducted in the absence of any commercial or financial relationships that could be construed as a potential conflict of interest.
