# Supplementary material for: Outcome of Patients With Esophageal Atresia and Very Low Birth Weight (≤ 1,500 g)
Source: Front Pediatr. 2020 Nov 17;8:587285. doi: 10.3389/fped.2020.587285 (PMC7705242; doi:10.3389/fped.2020.587285)
Supplement: Supplementary file 1 [file Data_Sheet_1.PDF]

LMU

KLINIKUM
DER UNIVERSITÄT MÜNCHEN

KINDERCHIRURGISCHE KLINIK
UND POLIKLINIK
IM DR. v. HAUNERSCHEN KINDERSPITAL

Direktor: Prof. Dr. med. D. v. Schweinitz

Studie zum

# Einfluss des operativen Vorgehens auf das Outcome von Patienten mit Ösophagusatresie und einem Geburtsgewicht unter 1500 g.

## Fragebogen

## Teil I: Allgemeine Fragen zu Patient und Operation

| | | |
|---|---|---|
| **Allgemein** | Allgemeine Informationen zu Ihrem Kind. Teile der Informationen finden sich in den Arztbriefen oder im Gelben Untersuchungsheft. Es wäre hilfreich, wenn Sie die anonymisierten Arztbriefe als Kopie anfügen könnten. | |
| Geboren am | Monat:_____ Jahr: _____ | Geschlecht ☐ weiblich<br>☐ männlich |
| Geburtsgewicht | Gramm: _____ | |
| | | |
| **Diagnosen** | Informationen zur Diagnose finden sich in den Arztbriefen. Es wäre hilfreich, wenn Sie die anonymisierten Arztbriefe als Kopie anfügen könnten. | |
| Ösophagusatresie | Vogt ☐ Typ I ☐ Typ II ☐ Typ IIIa ☐ Typ IIIb ☐ Typ IIIc ☐ H-Fistel | |
| | Long gap ☐ ja ☐ nein ☐ unbekannt | |
| Herzfehler | Welcher:_______________ | Hämodynamisch relevant ☐ ja ☐ nein ☐ unbekannt |
| Syndrom | ☐ ja, welches:_________________________ ☐ nein ☐ unbekannt | |
| Fehlbildungen | ☐ ja, welche: _________________________ ☐ nein ☐ unbekannt | |
| Hirnblutung | ☐ Grad I ☐ Grad II ☐ Grad III ☐ Grad IV ☐ nein ☐ unbekannt | |
| | | |
| **Operation** | Diese Informationen können Sie den Operationsberichten oder Arztbriefen entnehmen. Außerdem wäre es hilfreich, wenn Sie anonymisierte Operationsbericht und Arztbriefe als Kopie anfügen könnten. | |
| Vor der Operation beatmet | ☐ ja ☐ nein ☐ unbekannt | |
| Fistelverschluss | im Alter von _____ Tagen<br>Gastrostoma ☐ ja ☐ nein ☐ unbekannt<br>Jejunostoma ☐ ja ☐ nein ☐ unbekannt<br>Collare Fistel ☐ ja ☐ nein ☐ unbekannt | |

| | |
|---|---|
| Anastomose des Ösophagus | Zusammen mit dem primären Fistelverschluss ☐ ja ☐ nein ☐ unbekannt<br><br>Sollte die Anastomose erst in einer zweiten Operation genäht worden sein<br>im Alter von _____ Tagen<br>bei einem Gewicht von _____ Gramm |
| Operationen bis 6. Lebensmonat | ☐ eine ☐ zwei ☐ drei ☐ mehr als drei ☐ unbekannt |
| Beatmungsdauer | _____ Tage |
| Komplikationen | Anastomoseninsuffizienz ☐ ja ☐ nein ☐ unbekannt<br>Re-Fistel ☐ ja ☐ nein ☐ unbekannt<br>Anastomosenstenose ☐ ja ☐ nein ☐ unbekannt<br>Sonstige: _____ |
| Bougierungen | ☐ keine ☐ weniger als drei ☐ mehr als drei ☐ unbekannt<br>☐ vor dem 2. Lebensjahr ☐ nach dem 2. Lebensjahr |
| Gastroösophagealer Reflux | ☐ symptomatisch ☐ nicht symptomatisch ☐ unbekannt<br>☐ Säureblocker ☐ unbekannt<br>☐ Fundoplikatio im Alter von Monaten: _____ ☐ unbekannt<br>☐ aktuell Refluxbeschwerden ☐ keine Beschwerden |
| Schluckstörungen | Siehe separaten Bogen |
| Aktuelle Medikamente | _____<br>_____<br>_____ |
| Aktuelles Gewicht | _____ kg |

## II. Teil: Dysphagie Fragebogen

| | |
|---|---|
| 1) Es ist schwierig mit dem Schluckproblem meines Kindes umzugehen. | stimmt nicht stimmt völlig<br>0_____________________100 |
| 2) Das Schluckproblem unseres Kindes beeinflusst unser alltägliches Leben. | stimmt nicht stimmt völlig<br>0_____________________100 |
| 3) Mein Kind isst langsamer als andere Kinder | stimmt nicht stimmt völlig<br>0_____________________100 |
| 4) Es dauert eine Ewigkeit bis mein Kind mit einer Mahlzeit fertig ist. | stimmt nicht stimmt völlig<br>0_____________________100 |

| | |
|---|---|
| 5) Mein Kind kann das Essen nicht geniessen. | stimmt nicht stimmt völlig<br>0______________________________________100 |
| 6) Mein Kind möchte wegen des Schluckproblems nicht mehr essen. | stimmt nicht stimmt völlig<br>0______________________________________100 |
| 7) Für mein Kind ist es schwierig Nahrungsmittel zu finden, die es gerne isst. | stimmt nicht stimmt völlig<br>0______________________________________100 |
| 8) Es belastet unsere Familie Essen für mein Kind zu finden. | stimmt nicht stimmt völlig<br>0______________________________________100 |
| 9) Ich habe Angst, mein Kind könnte beim Essen fester Speisen ersticken. | stimmt nicht stimmt völlig<br>0______________________________________100 |
| 10) Ich habe Angst davor, dass mein Kind Lungenentzündungen bekommt | stimmt nicht stimmt völlig<br>0______________________________________100 |
| 12) Ich habe Angst, mein Kind könnte jederzeit ersticken. | stimmt nicht stimmt völlig<br>0______________________________________100 |
| 13) Es ist sehr anstrengend, das Essverhalten meines Kindes so streng beobachten zu müssen. | stimmt nicht stimmt völlig<br>0______________________________________100 |
| 14) Ich bin wegen der Schluckprobleme meines Kindes frustiert. | stimmt nicht stimmt völlig<br>0______________________________________100 |
| 15) Ich fühle mich wegen der Schluckprobleme meines Kindes entmutigt. | stimmt nicht stimmt völlig<br>0______________________________________100 |
| 16) Ich werde ungeduldig, wenn ich mich um die Schluckprobleme meines Kindes kümmern muss. | stimmt nicht stimmt völlig<br>0______________________________________100 |
| 16) Ich werde ungeduldig, wenn ich mich um die Schluckprobleme meines Kindes kümmern muss. | stimmt nicht stimmt völlig<br>0______________________________________100 |
| 17) Unsere Familienfreizeit Aktivitäten haben sich wegen der Schluckprobleme unsere Kindes geändert. | stimmt nicht stimmt völlig<br>0______________________________________100 |
| 18) Große Feste mit der Familie oder Freunden sind aufgrund der Schluckprobleme unseres Kindes schwierig. | stimmt nicht stimmt völlig<br>0______________________________________100 |

| | |
|---|---|
| 19) Es ist schwierig mit meinem Kind wegen der Schluckprobleme auswärts essen zu gehen. | stimmt nicht stimmt völlig<br>0________________________________________100 |
| 21) Mein Kind hustet. | stimmt nicht stimmt völlig<br>0________________________________________100 |
| 21) Mein Kind würgt/verschluckt sich wenn es feste Nahrung isst. | stimmt nicht stimmt völlig<br>0________________________________________100 |
| 22) Mein Kind würgt/verschluckt sich wenn es trinkt. | Stimmt nicht stimmt völlig<br>0________________________________________100 |
| 23) Mein Kind hat zähen Speichel oder eitriges Sekret. | stimmt nicht stimmt völlig<br>0________________________________________100 |
| 24) Mein Kind hat viel Speichel oder eitriges Sekret. | stimmt nicht stimmt völlig<br>0________________________________________100 |
| 25) Mein Kind muss würgen. | stimmt nicht stimmt völlig<br>0________________________________________100 |
| 26) Mein Kind hat Schwierigkeiten beim Kauen. | stimmt nicht stimmt völlig<br>0________________________________________100 |
| 27) Mein Kind muss sich häufig räuspern. | stimmt nicht stimmt völlig<br>0________________________________________100 |
| 28) Mein Kind hat Steckenbleiber im Hals. | stimmt nicht stimmt völlig<br>0________________________________________100 |
| 29) Mein Kind hat Steckenbleiber im Mund. | stimmt nicht stimmt völlig<br>0________________________________________100 |
| 30) Mein Kind speichelt Nahrungsmittel oder Flüssigkeit. | stimmt nicht stimmt völlig<br>0________________________________________100 |
| 31) Bei meinem Kind kommt manchmal Nahrung oder Flüssigkeit aus der Nase. | stimmt nicht stimmt völlig<br>0________________________________________100 |
| 32) Mein Kind hustet Nahrungsmittel oder Flüssigkeit / räuspert sich wenn sie im Hals stecken bleiben | stimmt nicht stimmt völlig<br>0________________________________________100 |
| 33) Die Schluckbeschwerden machen die soziale Interaktion schwierig für mein Kind. | stimmt nicht stimmt völlig<br>0________________________________________100 |

# III. Teil: KIDSCREEN, Erfassung der gesundheitsbezogene Lebensqualität

| **I. Gesundheit und Bewegung** | |
|---|---|
| 1.) Was denken Sie: Wie würde Ihr Kind seine Gesundheit im Allgemeinen beschreiben? | ☐ ausgezeichnet ☐ sehr gut ☐ gut<br>☐ weniger gut ☐ schlecht |
| 2.) Hat sich Ihr Kind in den letzten Wochen fit und wohl gefühlt? | ☐ überhaupt nicht ☐ ein wenig ☐ mittelmäßig<br>☐ ziemlich ☐ sehr |
| 3.) Hat sich Ihr Kind in den letzten Wochen viel bewegt (z.B. beim Rennen, Klettern, Fahrradfahren)? | ☐ überhaupt nicht ☐ ein wenig ☐ mittelmäßig<br>☐ ziemlich ☐ sehr |
| 4.) Konnte Ihr Kind in den letzten Wochen gut rennen? | ☐ überhaupt nicht ☐ ein wenig ☐ mittelmäßig<br>☐ ziemlich ☐ sehr |
| 5.) Ist Ihr Kind in den letzten Wochen voller Energie gewesen? | ☐ nie ☐ selten ☐ manchmal<br>☐ oft ☐ immer |
| **II. Gefühle und Stimmung** | |
| 1.) Hatte Ihr Kind das Gefühl, dass ihm sein Leben in den letzten Wochen gefällt? | ☐ überhaupt nicht ☐ ein wenig ☐ mittelmäßig<br>☐ ziemlich ☐ sehr |
| 2.) Hat Ihr Kind in den letzten Wochen gute Laune gehabt? | ☐ nie ☐ selten ☐ manchmal<br>☐ oft ☐ immer |
| 3.) Hat Ihr Kind Spaß gehabt? | ☐ nie ☐ selten ☐ manchmal<br>☐ oft ☐ immer |
| 4.) Hat sich Ihr Kind traurig gefühlt? | ☐ nie ☐ selten ☐ manchmal<br>☐ oft ☐ immer |
| 5.) Hat Ihr Kind sich so schlecht gefühlt, dass es gar nichts machen wollte? | ☐ nie ☐ selten ☐ manchmal<br>☐ oft ☐ immer |
| 6.) Hat Ihr Kind sich einsam gefühlt? | ☐ nie ☐ selten ☐ manchmal<br>☐ oft ☐ immer |
| 7.) Ist Ihr Kind zufrieden gewesen, so wie es ist? | ☐ nie ☐ selten ☐ manchmal<br>☐ oft ☐ immer |
| **III. Familie und Freizeit** | |
| 1.) Hat Ihr Kind in den letzten Wochen genug Zeit für sich selbst gehabt? | ☐ nie ☐ selten ☐ manchmal<br>☐ oft ☐ immer |
| 2.) Konnte Ihr Kind in den letzten Wochen in seiner Freizeit die Dinge machen, die es tun wollte? | ☐ nie ☐ selten ☐ manchmal<br>☐ oft ☐ immer |
| 3.) Hatte Ihr Kind in den letzten Wochen das Gefühl, dass seine Eltern genug Zeit für es hatten? | ☐ nie ☐ selten ☐ manchmal<br>☐ oft ☐ immer |
| 4.) Hat sich Ihr Kind in den letzten Wochen durch seine Mutter /seinen Vater gerecht behandelt gefühlt? | ☐ nie ☐ selten ☐ manchmal<br>☐ oft ☐ immer |
| 5.) Konnte Ihr Kind in den letzten Wochen mit seinen Eltern reden, wenn es wollte? | ☐ nie ☐ selten ☐ manchmal<br>☐ oft ☐ immer |
| 6.) Hat Ihr Kind in den letzten Wochen genug Geld gehabt, um das Gleiche zu machen wie seine Freunde? | ☐ nie ☐ selten ☐ manchmal<br>☐ oft ☐ immer |
| 7.) Hatte Ihr Kind in den letzten Wochen genug Geld für die Sachen, die es braucht? | ☐ nie ☐ selten ☐ manchmal<br>☐ oft ☐ immer |

| | |
|---|---|
| **IV. Freunde** | |
| 1.) Hat Ihr Kind in den letzten Wochen Zeit mit seinen Freunden verbracht? | nie selten manchmal<br>oft immer |
| 2.) Hat Ihr Kind in den letzten Wochen mit seinen Freunden Spaß gehabt? | nie selten manchmal<br>oft immer |
| 3.) Haben Ihr Kind und seine Freunde sich gegenseitig geholfen? | nie selten manchmal<br>oft immer |
| 4.) Hat Ihr Kind sich in den letzten Wochen auf seine Freunde verlassen können? | nie selten manchmal<br>oft immer |
| **V. Schule und Lernen** | |
| 1.) Ist Ihr Kind in den letzten Wochen in der Schule glücklich gewesen? | überhaupt nicht ein wenig mittelmäßig<br>ziemlich sehr |
| 2.) Ist Ihr Kind in den letzten Wochen in der Schule gut zurechtgekommen? | überhaupt nicht ein wenig mittelmäßig<br>ziemlich sehr |
| 3.) Konnte Ihr Kind in den letzten Wochen gut aufpassen? | nie selten manchmal<br>oft immer |
| 4.) Ist Ihr Kind in den letzten Wochen gut mit seinen Lehrerinnen und Lehrern ausgekommen? | |
