# Supplementary material for: Outcome of Patients With Esophageal Atresia and Very Low Birth Weight (≤ 1,500 g)
Source: Front Pediatr. 2020 Nov 17;8:587285. doi: 10.3389/fped.2020.587285 (PMC7705242; doi:10.3389/fped.2020.587285)
Supplement: Supplementary file 2 [file Table_1.DOCX]

Supplement 2: Distribution of patients over the time-period

| ***Year*** | ***n (% of all)*** | ***ELBW*** | ***Primary repair (% of n)*** | ***AI*** | ***RF*** | ***AS*** |
| --- | --- | --- | --- | --- | --- | --- |
| ***1988–1990*** | *1 (2%)* | *0* | *0 (0%)* | *unknown* | *unknown* | *unknown* |
| ***1991–1995*** | *2 (4%)* | *0* | *2 (100%)* | *unknown* | *unknown* | *1* |
| ***1996–2000*** | *4 (8%)* | *3* | *2 (50%)* | *1* | *1* | *2* |
| ***2001–2005*** | *7 (15%)* | *2* | *5 (71%)* | *0* | *1* | *5* |
| ***2006–2010*** | *6 (12%)* | *1* | *2 (33%)* | *1* | *1* | *3* |
| ***2011–2015*** | *11 (23%)* | *1* | *4 (36%)* | *3* | *1* | *6* |
| ***2016–2019*** | *18 (38%)* | *3* | *10 (55%)* | *4* | *4* | *7* |

*Time-period was clustered in five years. All patients born and treated in a time-cluster are listed under n. The percentage of primary repair refers to n.*
